# Remotely Delivered Behavioral Weight Loss Intervention Using an Ad Libitum Plant-Based Diet: Pilot Acceptability, Feasibility, and Preliminary Results

**DOI:** 10.2196/37414

**Published:** 2022-06-23

**Authors:** Christina Chwyl, Nicholas Wright, Gabrielle M Turner-McGrievy, Meghan L Butryn, Evan M Forman

**Affiliations:** 1 Center for Weight, Eating, and Lifestyle Sciences Drexel University Philadelphia, PA United States; 2 Royal New Zealand College of General Practitioners Wellington New Zealand; 3 Department of Health Promotion, Education, and Behavior Arnold School of Public Health Columbia, SC United States

**Keywords:** vegetarian diet, vegan diet, overweight, eHealth, behavioral intervention

## Abstract

**Background:**

Many traditional lifestyle interventions use calorie prescriptions, but most individuals have difficulty sustaining calorie tracking and thus weight loss. In contrast, whole food plant-based diets (WFPBDs) have previously shown significant weight loss without this issue. However, most WFPBD interventions are face-to-face and time-intensive, and do not leverage gold standard behavioral strategies for health behavior change.

**Objective:**

This open pilot trial was the first to evaluate the feasibility of a fully featured, remotely delivered behavioral weight loss intervention using an ad libitum WFPBD.

**Methods:**

Over 12 weeks, participants (N=15) with overweight or obesity received a newly designed program that integrated behavioral weight loss and a WFPBD prescription via weekly web-based modules and brief phone coaching calls. Assessments were performed at baseline, midtreatment (6 weeks), and after treatment (12 weeks).

**Results:**

The intervention was rated as highly acceptable (mean 4.40 out of 5, SE 0.18), and attrition was low (6.7%). In all, intention-to-treat analyses revealed that 69% (10.4/15) of the participants lost 5% of their weight (mean –5.89, SE 0.68 kg). Predefined benchmarks for quality of life were met.

**Conclusions:**

A pilot digital behavioral weight loss intervention with a non–energy-restricted WFPBD was feasible, and the mean acceptability was high. Minimal contact time (80-150 minutes of study interventionist time per participant over 12 weeks) led to clinically relevant weight loss and dietary adherence for most participants (10.4/15, 69% and 11.8/15, 79%, respectively), and quality of life improvements (reliable change indices >1.53). We hope that this work will serve as a springboard for future larger scale randomized controlled studies evaluating the efficacy of such programs for weight loss, dietary change, and quality of life.

**Trial Registration:**

ClinicalTrials.gov NCT04892030; https://clinicaltrials.gov/ct2/show/NCT04892030

## Introduction

### Background

Excess weight is a leading cause of death in industrialized countries and contributes to a wide range of health issues, including cardiovascular disease, type 2 diabetes, risk of certain cancers, and early mortality [1-4]. Standard approaches to weight loss rely on calorie prescriptions to achieve negative energy balance [5]. Calorie prescriptions, in conjunction with the provision of psychological and behavioral strategies (eg, goal setting, stimulus control, self-monitoring, problem solving, and cognitive restructuring) to facilitate lifestyle modification, are considered the current gold standard behavioral weight loss treatments (S-BTs) [6]. S-BTs produce, on average, 5% to 8% of body weight loss following intensive, year-long intervention [5]. However, one-third of individuals do not lose clinically significant levels of weight, and one-third of initial weight lost is regained in the year after treatment, with continued weight regain thereafter [7].

Overall, 2 core limitations (reliance on calorie tracking and lack of appetite control optimization) may contribute to the suboptimal outcomes of S-BT. S-BTs rely on meticulous dietary self-monitoring (ie, tracking everything consumed), which is considered the cornerstone of treatment success [5]. Indeed, thorough dietary self-monitoring is one of the strongest identified predictors of weight loss and maintenance outcomes following S-BT [8-10]. However, many participants find calorie tracking to be unappealing and arduous [11,12] and are unable to track their calorie intake consistently, accurately, and in the long term [11,13-15]. Another limitation of S-BT is that it is not optimized to address the increase in appetite that individuals face when losing weight [16]. That is, when individuals lose weight, a host of biological adaptations occur, including unfavorable changes to appetite that serve to guard against fat loss [16,17]. However, although appetite is recognized to play a critical role in governing energy intake and driving suboptimal weight loss and maintenance outcomes [18], S-BTs are not optimized to address the increase in appetite that individuals face during weight loss.

In contrast, a whole food plant-based diet (WFPBD) can produce clinically significant weight loss, health, and quality of life improvements in the absence of calorie tracking [19-22]. WFPBDs include vegetables, fruits, legumes, starches, and whole grains in minimally processed forms. This maximizes nutrient-dense, low–energy density foods, while limiting energy-dense foods (eg, processed foods and animal products). Decreasing dietary energy density with ad libitum consumption can result in weight loss while sating appetite [23-25], potentially by reducing passive intake of high-calorie or high-fat foods [26] by increasing the volume of food that cues fullness [27] or owing to the satiating effects of a high-fiber diet low in energy density [28]. Indeed, WFPBDs have been found to significantly decrease ad libitum consumption compared with other diets. For example, individuals assigned to eat WFPBD meals ad libitum ate, on average, 689 calories per day fewer than those assigned to eat animal-based, low-carbohydrate meals ad libitum [24]. Plant-based diets also merit consideration for health and environmental benefits. Regarding physical health, WFPBDs are robustly associated with reduced risk of chronic reduction, including reduced rates of cardiovascular risk [21,29,30], hypertension [21], type 2 diabetes [31], and certain cancers [32]. Regarding the environment, plant-based foods are superior to animal products in terms of land use, freshwater use, land acidification, and greenhouse gas emissions [33].

Despite the potential of WFPBDs for health and weight loss, existing interventions using WFPBDs have 2 key limitations. First, the vast majority of WFPBD interventions are conducted in-person, and delivery requires significant time of participants and highly trained professionals [7,20] (for an exception, see the study by Turner-McGrievy et al [34]), of which there is a shortage [35,36]. In contrast, remotely delivered weight loss interventions, especially those with minimal human support, have a higher potential for dissemination and can produce clinically significant weight loss when engagement (eg, interactivity) and health behavior change (eg, accountability and self-monitoring) features are included [15,37-39]. In addition, although gold standard approaches to weight loss incorporate psychological and behavioral strategies to facilitate lifestyle modification (eg, stimulus control and regular self-weighing), most WFPBD interventions have provided nutrition information alone [20,40]. This likely limits their efficacy, as behavioral and psychological skills are known to improve the efficacy of weight loss interventions [41], and simply knowing what to do to lose weight does not necessarily translate into behavior change, as demonstrated by the superior efficacy of S-BT to psychoeducation alone [42].

### Objectives

This open trial pilot study addressed these limitations by evaluating the feasibility of a remotely delivered digital behavioral weight loss intervention using an ad libitum WFPBD. The intervention was designed with accessibility in mind and thus was remotely delivered and required minimal time from the participants and clinicians. The intervention consisted of two core components: (1) weekly web-based modules delivering WFPBD nutrition counseling and gold standard behavioral weight loss strategies and (2) one-on-one, 10- to 15-minute phone *coaching* calls, with a study interventionist for most weeks. The primary aim was to evaluate recruitment feasibility and acceptability. The secondary aim was to evaluate the preliminary effect of the intervention on weight loss and dietary adherence. As an exploratory aim, we evaluated the preliminary impact of the intervention on the quality of life.

## Methods

### Participants

Adult men and women with overweight or obesity (BMI ≥25 kg/m^2^), aged 18 to 75 years, and residing in the United States were eligible as part of larger recruitment efforts for the ongoing weight loss trials at the Drexel University Center for Weight, Eating and Lifestyle Science (WELL Center). Recruitment for other weight loss trials in the center occurred through advertisements at radio stations and in newspapers and social media posts. Notably, no advertising specific to this study was conducted. Individuals interested in participating in a weight loss study were contacted via phone, and preliminary eligibility for weight loss trials in the WELL Center was assessed. Depending on the enrollment status of the other ongoing trials, individuals were provided preliminary information about this study and could elect to be screened for it. In addition, individuals ineligible for other trials in the WELL Center could elect to be screened for this study. Trained research staff then conducted a preliminary screening for this study. Approximately 0 to 2.5 months later, the first author (CC) contacted interested participants to discuss full eligibility. Full eligibility was assessed by phone, and baseline assessments were scheduled for those who were interested and eligible. Figure S1 in Multimedia Appendix 1 details participant flow and reasons for exclusion. Of the individuals who underwent preliminary screening for this study (N=86), the reasons for exclusion included lack of interest in following the study diet (25/86, 29%), >5% weight loss in the past 3 months (10/86, 12%), medical condition influencing weight or appetite (8/86, 9%), ongoing participation in another weight loss trial underway at the center (6/86, 7%), eating disorder pathology (3/86, 3%), poor English comprehension (3/86, 3%), already following the study diet (2/86, 2%), inability to attend appointments (2/86, 2%), BMI <25 (2/86, 2%), inability to follow the study diet (1/86, 1%), and consumption of medication known to cause weight gain (1/86, 1%).

The participants were recruited between November 2020 and February 2021. Given the novelty of the intervention, before the pilot trial, 7 participants were enrolled in a pretest of the intervention between December 2020 and February 2021. Following the pretest, refinements to the intervention were made, including streamlining content, addressing common confusion about the WFPBD earlier on, and adding more structured questions and goal setting to the phone coaching calls. Table S1 in Multimedia Appendix 1 presents a list of intervention refinements and the rationale for each. The refined intervention was then delivered to a set of 15 participants in the pilot trial, starting between January and February 2021 and ending between March and April 2021. Because this was a pilot feasibility study, a power analysis was not performed [43]. We aimed for a sample of 14 participants, because we deemed this sample size sufficient to evaluate feasibility and acceptability. Notably, this sample size is consistent with prior internet-delivered weight loss pilot interventions [44]. Exclusion criteria were the use of medications for weight loss, ≥5% weight loss in the past 3 months, current or planned pregnancy within the study period, bariatric surgery history, currently following a WFPBD, diagnosis of a serious medical or psychiatric condition influencing weight or appetite, high substance use, and eating pathology (ie, binge eating disorder diagnosis or subthreshold loss-of-control eating or compensatory behaviors).

### Study Design

The Template for Intervention Description and Replication checklist and guide was used to inform the description of the intervention [45]. Reporting followed the guidelines of the CONSORT (Consolidated Standards of Reporting Trials) extension to pilot and feasibility trials (excluding items specific to randomization) [46]. The intervention comprised two components: (1) weekly web-based modules delivering behavioral weight loss strategies and WFPBD nutrition psychoeducation and (2) brief 10- to 15-minute *coaching* calls with a study interventionist.

### Dietary Prescription

Participants were prescribed a WFPBD that promoted the intake of fruits, vegetables, starches, legumes, and whole grains [47]. Participants were encouraged to avoid processed foods, refined oils, and animal products and to minimize the consumption of high-fat plant-based foods. An adapted *traffic-light* diet chart (Figure S2 in Multimedia Appendix 1 [47]) outlined foods to eat, limit, and avoid. The participants were advised to eat until satiation (without restricting energy intake). Consistent with past WFPBD interventions and to ensure nutritional adequacy, participants were instructed to consume 50 μg of vitamin B12 supplements daily [47,48]. The participants were asked to purchase vitamin B12 supplements. If doing so imposed a financial burden, participants were mailed vitamin B12 supplements.

### Behavioral Intervention

The behavioral or psychological strategies used in the intervention were developed based on existing gold S-BT [49], evidence-based behavioral change techniques (BCTs) [50], and social cognitive theory [51]. Content specific to WFPBD was primarily adapted from the BROAD study [47]. The intervention was also informed by feedback from 7 participants who experienced the preliminary *beta* version of the program (in particular, participant feedback resulted in the addition of mobile-friendly and printable PDFs of key materials, links to transcripts of videos, individualized weight goals, streamlined modules, more detailed information on the traffic-light diet chart provided in weeks 1 and 2, increased representation of body sizes in intervention content, a Google Classrooms tutorial, and guided inquiry related to nonadherence to the WFPBD). To inform intervention replication, Table S2 in Multimedia Appendix 1 presents a list of the BCTs used in this study according to the BCT taxonomy by Michie et al [50], which includes 93 individual behavior techniques grouped into 16 superordinate clusters. The intervention included a variety of BCTs (14 BCT clusters and 31 specific techniques).

### Modules

The weekly web-based modules delivered in the intervention were created using a Google Slide add-on (Pear Deck) that enabled the integration of interactive questions were hosted on a popular e-learning platform (Google Classroom). Content was presented through a combination of audio, text, images, and interactive elements (eg, written reflection prompts, draggable questions, and *Knowledge Check* quizzes). The materials were adapted from existing, successful, and in-person behavioral weight loss treatments. Specifically, WFPBD nutrition counseling content was based primarily on materials delivered in the BROAD study [47] and featured materials on energy density [25], food cravings [52], and nutrition psychoeducation [53,54]. Psychological and behavioral skills were similar to those used in the Diabetes Prevention Program protocols [55] and past successful behavioral weight loss treatments [56] and included core behavioral strategies for weight loss and maintenance, such as stimulus control [49], problem solving [49], habit formation [57], and relapse prevention [58]. Content was divided into three parts: (1) transition (weeks 1 and 2, in which participants were asked to begin transitioning to a WFPBD), (2) change (weeks 3-6, in which participants were asked to fully adopt a WFPBD), and (3) sustain (weeks 7-12, in which participants received behavioral and psychological skills aimed at facilitating long-term weight maintenance). The modules were designed to take between 10 and 30 minutes to complete. Table S3 in Multimedia Appendix 1 presents an outline of the weekly intervention content. Following each module, to solidify learning, participants completed weekly worksheets (*Put into Practice Assignments*).

Each week, a new module was made available. The participants accessed each module individually and completed it at their own pace. Participants could complete modules at any time within the assigned week but were encouraged to set aside a consistent time to view the intervention content each week. Each week, participants had access to additional recipes, optional further reading, and examples of people who had successfully adopted a WFPBD lifestyle. We included cooking and educational resources from a diverse set of people in terms of age, race, gender, and cultural background. If participants did not complete the module, up to 2 follow-up emails were sent or reminder calls, made.

### Phone Coaching

To facilitate dietary adherence and provide accountability and individualized feedback, key components of successful weight loss interventions [50], 9 to 11 one-on-one, 10- to 15-minute phone check-in calls were scheduled. The participants were permitted to miss up to 2 coaching calls in the event of holidays or sickness, and the final coaching call was optional. Phone coaching was conducted by the primary investigator, a clinical psychology doctoral student with training and prior experience in delivering behavioral weight loss. Before the study, the primary investigator received day-long training in behavioral weight loss from experienced, advanced practitioners and received weekly individual supervision on effective delivery of behavioral weight loss. Calls consisted of positive reinforcement, problem-solving support, and motivational support, in line with a motivational interviewing approach [59]. Figure S3 in Multimedia Appendix 1 details the full phone coaching protocol.

### Weekly Weighing

Given the research on the advantages of frequent self-weighing [60], each week, participants entered their weight into a web-based spreadsheet that auto-populated a graph to visually depict progress. Automated emails prompted participants to enter their weight.

### Measures

Assessments were performed at baseline, midtreatment (6 weeks), and after treatment (12 weeks) and were conducted remotely via Zoom (Zoom Video Communications, Inc). Informed consent was obtained at the baseline assessment, and participants were provided a tutorial on how to complete the modules and use Google Classrooms. A compensation of US $10 for the midtreatment (6 weeks) and US $20 for the posttreatment (12 weeks) assessment was provided.

### Plant-Based Diet Familiarity

To contextualize our sample, we assessed plant-based diet history at baseline by asking participants to report whether they had eaten a plant-based diet in the past, for at least three months. Participants’ responses were coded into the following categories: previously vegan, vegetarian, pescatarian, flexitarian, or no prior plant-based diet familiarity (omnivorous).

### Acceptability

Acceptability was assessed after treatment with a questionnaire adapted from prior work [44] that asked participants to report how satisfied they were with the program, the degree to which they found the program helpful, and the likelihood that they would recommend it to family or friends using a Likert scale ranging from 1 (not at all) to 5 (very much). After treatment, participants reported how helpful they found each intervention component (phone check-ins, modules, Put into Practice Assignments, and weight charts). Items were averaged to calculate a composite acceptability score. The intervention was considered feasible if the mean acceptability ratings >4 out of 5 for at least 80% of the participants, a rate consistent with prior digital interventions [44]. To inform iterative development, following each module, participants rated the degree to which the module was helpful and engaging using the same scale. Modules with an average rating >4 out of 5 were considered acceptable.

### Feasibility

To evaluate feasibility, we examined the percentage of participants who were successfully retained in the study (defined as completing at least 10 of the 12 weekly modules and posttreatment assessment). The intervention was considered feasible if at least 80% of the participants met our retention criteria (completing at least 10 of the 12 modules as well as the posttreatment assessment), a retention rate consistent with previous brief digital interventions [39]. In addition, we examined the number of modules completed by the participants.

### Anthropometric Data

Participants self-weighed weekly and at the baseline, midtreatment (6 weeks), and posttreatment (12 weeks) assessments, following recommended guidelines, that is, instructed to weigh the first thing in the morning at least three times (a fourth if weights are discrepant by >0.2 lbs), in no or light clothes, and ensuring the scale is on a hard, flat surface. Weight was self-reported in pounds and was later converted to kilograms by the researchers. Self-reported weight following such guidelines generally have high accuracy [61,62]. Consistent with the weight losses observed in past interventions of a 12-week duration that were successfully delivered remotely with minimal human support [39,63,64], we considered weight loss to be suggestive of a preliminary impact if approximately 50% of our sample lost clinically meaningful levels of initial body weight (5% of initial body weight) [65].

### Dietary Adherence

At baseline, midtreatment (6 weeks), and after treatment (12 weeks), participants completed an adapted 18-item food frequency questionnaire aligned with the prescribed WFPBD traffic-light diet (Figure S2 in Multimedia Appendix 1 [47]) using a Likert scale ranging from 1 (rarely or never) to 6 (3 or more times per day). We examined the mean differences in consumption of green-, yellow-, and red-zone foods and overall dietary improvements (ie, weighted scores for the yellow- and red-zone foods subtracted from the green-zone foods). To weight scores, we multiplied responses in the ultragreen- and ultrared-zone categories by 3 and light green– and light red–zone food categories by 2. For interpretability, we transformed the scores to a 0 to 100 scale, with higher scores representing greater adherence. We considered dietary change to be meaningful and indicative of a preliminary impact if at least 80% of participants improved their dietary adherence score by at least 20 points. Adapted food frequency questionnaires have been found to be a valid method for capturing dietary intake across a wide range of omnivorous and plant-based diets [66].

### Quality of Life

Participants completed the 36-item Short-Form General Health Survey (SF-36) [67] at baseline, midtreatment (6 weeks), and after treatment (12 weeks). The SF-36 assesses 8 domains: physical functioning, limitations owing to physical health, pain, general health, energy or fatigue, social functioning, emotional well-being, and mental health. This measure has been shown to have good validity [68]. Scores were converted to a 0 to 100 scale, with higher scores indicating better functioning. This scale produces summaries of both physical health and mental health components. Owing to an error in survey creation, item 22 was not presented at baseline, although it was presented at midtreatment and after treatment. The omission of item 22 did not appear to change the results or adversely affect internal consistency. Internal consistency in our sample was excellent for physical health (α=.92) and good (α=.85) for the mental health component summary. Because the level of change in SF-36 scores that reflect a clinically significant improvement among weight loss samples is not yet well established, we calculated a reliable change index at the 0.20 level [69]. If the product exceeds a *z*-score of 1.28, reflecting 80% confidence, we consider our results to be suggestive of a preliminary impact.

### Statistical Analyses

Descriptive statistics were calculated for each outcome and process measure to evaluate whether benchmarks (specified a priori) thought to represent feasibility across several outcome measures were achieved (Table 1). Wilcoxon signed-rank tests were used to examine the preliminary impact of the intervention on weight loss, dietary change, and quality of life from before to after treatment. All variables are reported as the mean and SE of the mean or as frequencies. Analyses were conducted per-protocol (PP) and using the intention-to-treat (ITT) approach [55]. We imputed missing data (n=1 at midtreatment and after treatment) using multiple imputation procedures, a recommended approach for dealing with missing weight loss data [70]. We used SPSS (version 26) to conduct multiple imputations, specifying 5 generated data sets.

**Table 1:** Summary of intervention outcomes.

Outcome		Benchmark	Benchmark attainment
Feasibility		≥80% retained	93.3^a^
Program acceptability		≥80% acceptability ≥4 out of 5	77.3
Weight loss		≥50% achieving 5%	69.3^a^
Dietary adherence		≥80% changed diet by ≥20 scale points	78.7
**Quality of life, RCI^b^**			
	Physical health component summary	≥1.28	3.94^a^
	Mental health component summary	≥1.28	1.53^a^

^a^Outcomes that met or exceeded our benchmark. The results were reported from the intention-to-treat analyses.

^b^RCI: reliable change index.

### Ethics Approval

All aspects of the study design were specified a priori and the Drexel University Institutional Review Board approved this study (protocol number 2008008061). The study was registered at ClinicalTrials.gov on May 19, 2021 (identifier NCT04892030). All the participants provided written informed consent.

## Results

### Baseline Characteristics

A total of 15 individuals participated in the study. The sample was primarily White (11/15, 73%; 3/15, 20% Black or African American; 1/15, 7% South Asian), middle-aged (mean 52.67, SE 2.12 years), employed full- (11/15, 73%) or part-time (1/15, 7%), and married (10/15, 67%). All participants were female and identified as women. BMI ranged from 26.66 to 80.49 kg/m^2^ (median 35.25, SE 4.58 kg/m^2^). All participants endorsed at least some higher education: with 47% (7/15) reporting a postgraduate degree, 27% (4/15) reporting a college degree, 13% (2/15) reporting an associate degree, and 13% (2/15) reporting some college. Participants had variable prior experiences with plant-based eating: with 13% (2/15) having been flexitarian, 13% (2/15) having been pescatarian, 20% (3/15) having been vegetarian, and none having been vegan. There was no discernible pattern for income brackets, and 20% (3/15) of the participants chose not to answer.

### Summary of Results

**Figure 1:**
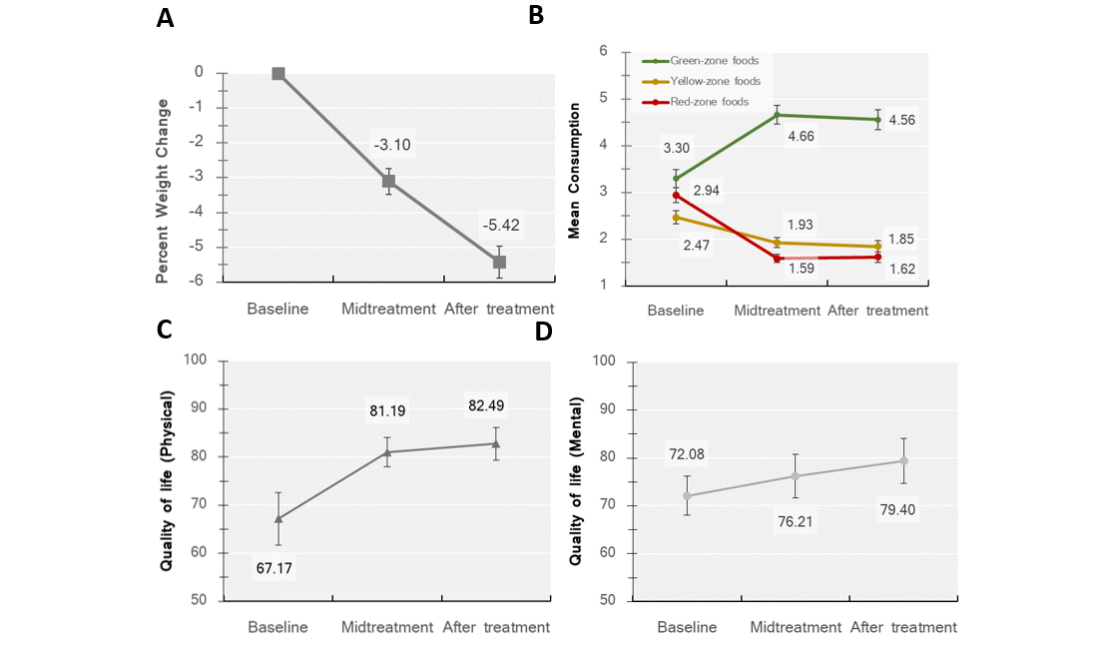
Outcomes at midtreatment and after treatment. Mean values for participants (N=15) at baseline, midtreatment (6 weeks), and after treatment (12 weeks) for (A) percent weight change, (B) diet change on the adapted food frequency questionnaire, (C) quality of life (physical component) as assessed by the 36-item Short-Form General Health Survey (SF-36), and (D) quality of life (mental component), as assessed by the SF-36. Error bars represent SE of the mean. Percent weight change and percent waist change, rather than percent weight loss and waist circumference loss, are depicted by the convention.

Table 1 summarizes the benchmark attainment for each outcome measure. Table 2 presents the descriptive statistics of each outcome measure at midtreatment and after treatment, separately. Figure 1 presents the outcomes at midtreatment and after treatment. Variables are reported as mean and SE or as frequency and percentage.

**Table 2:** Differences in outcome measures at midtreatment and after treatment.

				Baseline (0 weeks), mean (SE)		Midtreatment (6 weeks), mean (SE)		After treatment (12 weeks), mean (SE)		Change, mean (SE)
**Anthropometric measures**										
	Weight (kg)		113.95 (11.87)		104.67 (10.09)		102.03 (9.97)		−5.89 (0.68)	
**Self-report surveys**										
	**WFPBD FFQ^a^ (0-100)^b^**		56.24 (1.95)		82.35 (2.01)		81.35 (2.21)		25.17 (1.70)	
		Green zone (raw mean)	3.30 (0.19)		4.66 (0.20)		4.56 (0.21)		1.14 (0.14)	
		Yellow zone (raw mean)	2.47 (0.14)		1.93 (0.11)		1.85 (0.13)		−0.61 (0.11)	
		Red zone (raw mean)	2.94 (0.16)		1.59 (0.09)		1.62 (0.11)		−1.38 (0.12)	
	Quality of life (physical)		67.17 (5.45)		81.02 (3.08)		82.76 (3.38)		13.54 (3.96)	
	Quality of life (mental)		72.08 (4.07)		76.21 (4.52)		79.40 (4.73)		7.43 (4.78)	

^a^WFPBD FFQ: whole food plant-based diet food frequency questionnaire.

^b^The WFPBD FFQ transformed to a 0 to 100 scale. A score of 0 represented complete dietary nonadherence (ie, frequent intake of red and yellow zone foods and no or limited intake of green-zone foods), and a score of 100 represented complete adherence.

### Feasibility

The feasibility benchmark of retaining at least 80% of the participants was met (Table 1). A participant dropped out of treatment in week 2. Of the remaining participants, only 1.2% (2/168) modules were collectively missed. Participants received 8 to 10 coaching calls (80-150 minutes of coaching over the 12-week intervention period). Of the treatment completers, 14% (2/14) received 8 calls, 36% (5/14) received 9 calls, and 50% (7/14) received 10 calls. The reasons for missed calls included holidays and sickness.

### Acceptability

Acceptability was, on average, high (ITT: mean 4.40, SE 0.18; PP: mean 4.43, SE 0.18), although the percentage of participants attaining the program acceptability benchmark (ITT: mean 77.3%, SE 11.9%; PP: mean 73.3%, SE 26.7%) fell slightly below our goal of 80% attaining this benchmark (Table 1). Participants rated the weekly weigh-ins as most helpful (ITT: mean 4.53, SE 0.19; PP: mean 4.57, SE 0.17), followed by the web-based modules (ITT: mean 4.39, SE 0.21; PP: mean 4.43, SE 0.2), phone check-ins (ITT: mean 4.2, SE 0.29; PP: mean 4.21, SE 0.28) and Put into Practice Assignments (ITT: mean 3.96, SE 0.34; PP: mean 4, SE 0.31). All modules were rated as acceptable (≥4 out of 5) except for week 7, which presented content on common nutrition myths and advanced cooking techniques (ITT: mean 3.87, SE 0.27; PP: mean 3.89, SE 0.27) and week 8, which presented content on social support (ITT: mean 3.97, SE 0.23; PP: mean 3.96, SE 0.23). The highest rated module was week 11, which presented content on relapse prevention (ITT: mean 4.45, SE 0.19; PP: mean 4.42, SE 0.21), followed by week 9, which presented content on stress and emotional eating (ITT: mean 4.42, SE 0.21; PP: mean 4.46, SE 0.2).

### Weight Loss

The weight loss benchmark was attained (Table 1). Weight loss (ITT: mean 5.89, SE 0.68 kg; PP: mean 5.86, SE 0.73 kg) was observed before to after intervention (*z*=−3.41; P<.001; Cohen *d* 0.74).

### Dietary Change

The percentage of participants meeting the dietary adherence benchmark (ITT: 11.8/15, 79%; PP: 11/14, 79%) fell slightly short of our goal of 80% of participants meeting this benchmark (Table 1). Large dietary changes (ITT: mean 25.17, SE 1.70; PP: mean 25.2, SE 1.80) were observed from before to after treatment (*z*=−3.41; P<.001; Cohen *d* 1.55).

### Quality of Life

The quality of life benchmark was met (Table 1). Large changes in the physical health component summary of the SF-36 (ITT: mean 13.54, SE 3.96; PP: mean 13.48, SE 4.24) were observed before to after treatment (*z*=−3.24; P<.001; Cohen *d* 0.97). Small to medium changes (ITT: mean 7.43, SE 4.78; PP: mean 7.59, SE 5.13) were observed in the mental health component summary (*z*=−1.24; P=.22; Cohen *d* 0.45).

## Discussion

### Principal Findings and Comparison With Prior Work

This open pilot trial was the first to evaluate a remotely delivered ad libitum WFPBD behavioral weight loss intervention for adults with overweight or obesity, with minimal coaching support contact (80-150 minutes per patient over the course of 3 months). Our study was unique in that it integrated behavioral weight loss with an ad libitum WFPBD prescription that was remotely delivered. The results support the feasibility of the intervention and the preliminary impact of the intervention on weight loss and quality of life. The overall acceptability ratings were high, although they did not reach our acceptability benchmark (ITT: 77.3% vs the 80% prespecified). Similarly, dietary change over the course of the intervention was large (Cohen *d* 1.55), although dietary adherence did not reach the dietary adherence benchmark (ITT: 78.7% vs 80% prespecified). Thus, this study extends research on the feasibility and, potentially, the acceptability of a remotely delivered lower intensity format [20]. Our results support future studies examining the impact of interventions on weight loss, dietary adherence, and quality of life.

The results are promising given the need for more accessible and lower intensity weight loss treatments [71], especially those that do not require substantial participant time (eg, calorie tracking) or staff time (approximately 10-15 minutes weekly). In contrast, current gold standard behavioral treatments are expensive to deliver and require a workforce of expert clinicians, of which there is a shortage [72]. Therefore, if supported by further research, policy makers and practitioners could offer such a program to individuals seeking weight loss.

In contrast to traditional weight loss approaches that rely on calorie tracking to achieve a negative energy balance, the outcomes of our study were achieved with an ad libitum diet. Thus, our work extends the literature on weight loss approaches that rely on natural satiation mechanisms to achieve negative energy balance [25,73]. Although our results bear replication, the percentage of participants meeting the 5% weight loss threshold is similar to the level of weight loss achieved in other web- or app-based behavioral weight loss programs requiring food tracking and supplemented by remote human support [39,74]. In addition, clinically significant changes in diet and quality of life were observed in most participants. Thus, in contrast to prevailing weight loss approaches that produce weight loss to the extent that individuals track calories and adhere to specific calorie goals [5], WFPBDs do not require burdensome calorie tracking. Instead, they allow individuals to achieve negative energy balance through natural satiation mechanisms and may thus represent a promising alternative weight loss approach. Further research on behavioral weight loss treatments using ad libitum diets is critical, given the demand for non–calorie-tracking weight loss approaches [12,75] and given that calorie tracking noncompliance is prevalent and largely tracks weight regain [7,9] among individuals in weight loss programs that prescribe calorie goals.

Finally, this study was unique in its integration of WFPBD nutrition psychoeducation with behavioral and psychological principles for health behavior change [49,50,55]. Such strategies (eg, regular self-weighing and relapse prevention), as well as the inclusion of features shown to produce engagement and health behavior change in prior remotely delivered interventions (eg, interactivity, personalized elements, accountability, and self-monitoring), may facilitate efficacy [15,37,39]; however, this requires testing with a larger sample.

### Limitations

This study had several limitations. First, the observed enrollment rate of 27% is somewhat smaller than that observed in other trials at our center (33%-38%) [49,76]. Several factors may have resulted in a slightly lower enrollment yield rate, including the fact that participants were recruited from a generic pool of participants (vs the usual practice of advertising a specific study), interested individuals were contacted after up to a 2.5-month delay, and screenings for several weight loss trials occurred concurrently, leading some individuals to, instead, enroll in other trials. We anticipate that recruiting for a fully powered trial will be feasible, especially with study-specific advertisements.

Dietary acceptability also affected the enrollment rate; 29% (25/86) of individuals screened for this study were excluded owing to a lack of interest in following the WFPBD, suggesting that a WFPBD is not universally acceptable. Notably, conventional diets used in S-BT (which require meticulous calorie tracking) are likewise not universally acceptable [12] and may prevent individuals from enrolling in S-BT. In addition, many individuals enrolled in S-BT are unable to sustain calorie-tracking requirements, suggesting that conventional diets are not feasible over the long term for many [15]. Thus, although not universally acceptable, ad libitum WFPBDs may represent a viable alternative, especially for those who find calorie-tracking approaches unappealing or unsustainable. A promising future research direction is to evaluate recruitment feasibility for behavioral weight loss programs using standard versus ad libitum WFPBDs.

As this was an open pilot trial, results need to be replicated in larger samples, with a control condition and long-term follow-up period. Given that ad libitum WFPBD interventions rely on natural satiation mechanisms rather than calorie tracking to achieve negative energy balance, it is possible that a behavioral weight loss program using an ad libitum WFPBD may produce more sustained weight loss outcomes than behavioral weight loss interventions using a traditional calorie-prescribed diet [77]; however, this question requires empirical testing. Given the notorious challenge of obtaining accurate self-reports of dietary intake [11], future research should include more rigorous measures of dietary intake, such as 24-hour recalls administered by a registered dietician. The dietary adherence measure in this study was likely limited by retrospective bias (eg, reports over the past month) and may have been affected by participant characteristics (eg, those with higher conscientiousness or nutrition knowledge may have had higher accuracy).

Notably, our sample consisted of middle-aged women with higher than average educational attainment [78]. The degree to which our findings can be generalized to samples beyond those represented in this study is unknown. Our sample may have also had higher than average levels of motivation to lose weight or change their diet, including greater willingness to make substantive dietary changes. Future research would benefit from examining not only the efficacy of behavioral weight loss treatments using WFPBDs but also its effectiveness in community samples [47]. It is also important to note that this study was conducted during the COVID-19 pandemic, which may have influenced dietary behavior.

Finally, to assist in intervention streamlining and cost-effectiveness, future research would benefit from disentangling active treatment components from inert ones, examining the optimal dose of costly intervention components (ie, phone coaching) and exploring stepped care approaches (eg, providing phone coaching only to those who do not respond to modules alone or automated messages). Indeed, in this intervention, acceptability ratings for phone coaching were variable (mean 4.20, SE 0.29), with some participants reporting that phone coaching was an essential treatment component for them, while others reporting a desire for less frequent meetings.

### Conclusions

In sum, the results supported the feasibility of a 12-week remotely delivered intervention, prescribing a nonenergy-restricted WFPBD with minimal human contact (10-15 minutes most weeks). Feasibility was achieved, and the results support further research to evaluate efficacy for weight loss, dietary adherence, and quality of life. The program appears promising, given the need for more accessible alternatives to in-person and calorie tracking–based weight loss approaches. If supported by further research, such an intervention could be considered a frontline treatment, owing to the cost-effectiveness of digital treatments [74]; the limited time required of participants and staff; individuals’ preferences for lower intensity treatments [79]; the need for remotely delivered treatments, especially in light of the COVID-19 pandemic; and the wide-ranging health benefits of this dietary approach. This intervention might be of particular benefit to individuals who do not have access to in-person treatment, who do not benefit from S-BT, or who find calorie-tracking approaches to weight loss difficult to sustain. To build upon the current research, we are currently conducting a randomized controlled trial evaluating the efficacy of a remotely delivered behavioral weight loss intervention using an ad libitum WFPBD, in comparison with the current gold standard behavioral weight loss approach (S-BT).

## Appendix

Multimedia Appendix 1 Untitled.

## Abbreviations

BCT: behavioral change technique
CONSORT: Consolidated Standards of Reporting Trials
ITT: intention-to-treat
PP: per-protocol
S-BT: standard behavioral weight loss treatment
SF-36: 36-item Short-Form General Health Survey
WELL Center: Drexel University Center for Weight, Eating and Lifestyle Science
WFPBD: whole food plant-based diet
