# Supplementary material for: Remotely Delivered Behavioral Weight Loss Intervention Using an Ad Libitum Plant-Based Diet: Pilot Acceptability, Feasibility, and Preliminary Results
Source: JMIR Form Res. 2022 Jun 23;6(6):e37414. doi: 10.2196/37414 (PMC9264123; doi:10.2196/37414)
Supplement: Multimedia Appendix 1 [file formative_v6i6e37414_app1.docx]

Supplementary Materials

*Supplementary Figure S1*. Flow Diagram


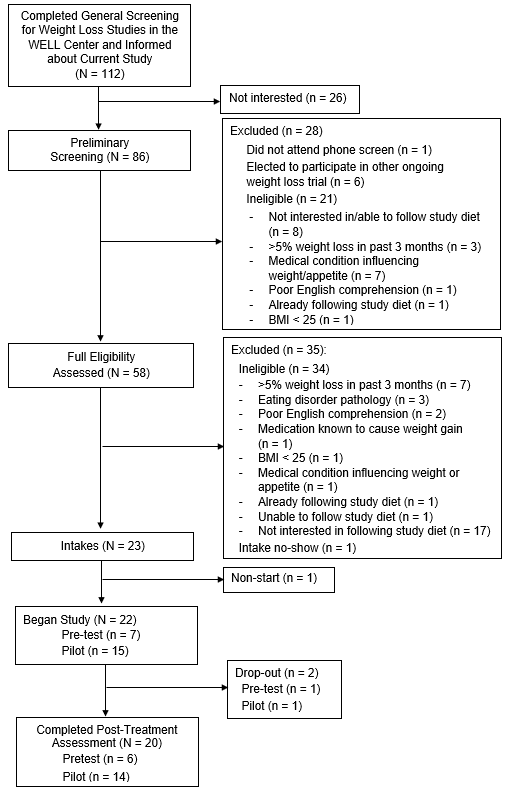


*Note.* Recruitment efforts occurred as part of larger recruitment efforts for ongoing weight loss trials at in the Weight Eating and Lifestyle Science (WELL) Center. Depending upon the recruitment status of the other weight loss trials in the WELL Center, individuals (*N* = 112) were informed about the present study and could elect to be screened for it. Medical exclusion criteria were insulin-dependent diabetes, head trauma, stroke, or a medical condition currently affecting weight or appetite (e.g., hypothyroidism, cancer).

*Supplementary Figure S2.* Traffic Light Diet Chart


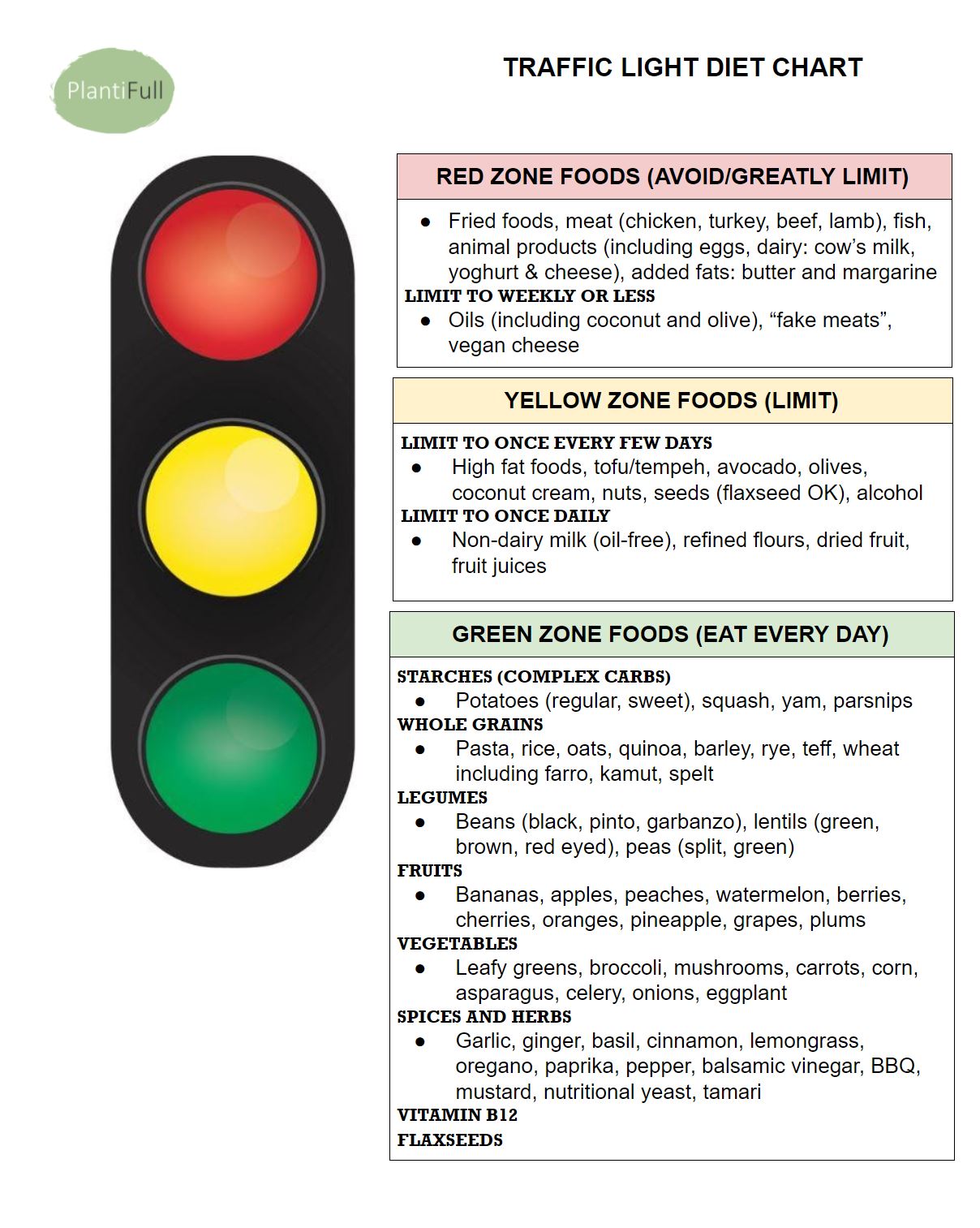


# *Supplementary Figure S3.* Appendix C: Phone Coaching Protocol

Phone Coaching Protocol Overview

The main goal of these coaching calls is to ensure that participants are adhering to the whole foods-plant based diet. In addition, phone coaching serves to promote adherence to the various components of the study (modules, worksheets, and weekly self-weighing). Coaches will begin calls by briefly reviewing with participants their weight graph:

*Script [If lost 1-2 pounds]*

- *Hi XXX. This is XXX calling from the XXX research project. Is now a good time to chat?*
- *It’s nice to have the chance to check-in with you again this week! How are you?*
- *Have any challenges arisen this past week with eating a whole foods plant-based diet? Have you been able to follow a whole foods plant-based diet?*
- *From your weight chart, it looks like you are right on track, and that you have lost XX pounds again this week. Way to go!*
- *Thank you for agreeing to participate in the research we really appreciate your efforts.*

*Script [If lost less than 1 pound, or gained weight]*

- *Hi XXX. It’s nice to have the chance to check-in with you again this week! How are you?*
- *From your weight chart, it looks like you have [lost/gained XX pounds this week]. Is there anything that has been making it difficult for you to eat a whole foods plant-based diet this past week? To what degree have you been able to follow a whole foods plant-based diet?*

When problem-solving with participants, be aware that there are several reasons that they might not be losing weight or adhering to a whole foods plant-based diet. Below, are potential reasons, as well as ways in which phone coaches can help problem solve for each. Ask them what they think is going on – try not to give too much advice here and just ask them to reflect on what’s happening.

*Participants think they are eating well*.

- Validate that this is a frustrating experience, to not have lost weight, despite feeling like they are eating well. Remind participants that we are here to support them and that a number of factors could be at play. Most likely, red zone foods are creeping into their diet, or have not actually been eliminated, or they’re trying to finish off those remaining foods. Ask participants for specific meals and snacks that they have been eating over the past week. Have participants check ingredients in any packaged foods that they have been eating. Encourage participants to eat simple, low-fat, whole foods and avoid packaged foods. If it is still unclear why participants are not losing weight, have them write out a sample meal plan. Ensure the sample meal plan adheres to the prescribed whole foods plant-based diet and is practical for them to follow. Get them to buy in to the idea of the ‘experiment’ and it only works if they do it properly – so just move the start date for them and get them to commit fully from the next week or the week after – when it suits them.

*Stress/time constraints*.

- Validate that prioritizing healthy eating and making large lifestyle changes is difficult. Ask participants why now is and is not a good time to make a large lifestyle change. Discuss simple, convenient meal options for participants, as well as strategies such as meal prepping, and stress reduction. Discuss ways in which to make their home environment as conducive to healthy eating as possible (e.g. having a dedicated shelf for whole, plant-based foods, talking with family to garner support, placing healthy foods on counter tops and at the front of the refrigerator).

*Lack of motivation and buy-in.*

- Validate that prioritizing healthy eating and making large lifestyle changes is difficult. Ask participants why now is vs. is not a good time to make a large lifestyle change. Adopt a “motivational interviewing approach.” Do not try to convince participants that they should adopt the whole foods plant-based diet. For example:
  - *Participant: Honestly, this has been really hard for me. I miss my usual foods. Social outings are awkward, and my friends make fun of me for ordering hummus and vegetables, and not eating wings with them like I usually do.*
  - *Phone coach: I can hear how frustrated you are. Switching to a whole foods plant-based diet is really difficult. It sounds like you’re encountering lots of challenges in your life, and that your friends in particular are not making this easy for you.*
  - *Participant: Yeah, for sure.*
  - *Phone coach: You know, dietary change is not for everyone, and it’s not always the right time for people to make these changes to their lifestyle. It’s possible that this isn’t the right time to make a lifestyle change for you.*
  - *Participant: Yeah, I mean it’s not the best time. But then again, when is it ever a good time to make a change.*
  - *Phone coach: Right, that’s a good point. The circumstances will never be ideal. Well, maybe you can treat these next XX weeks as an experiment. Maybe this is not the right lifestyle for you, and maybe it is. Your old foods will always be there for you to return to, right?”*
  - *Participant: “Yeah, that’s true. I think I can give this a go.”*

*Cravings.*

- Provide psychoeducation around cravings. Provide the analogy of going to a movie theatre. When we come out of the dark theatre, it takes awhile for our eyes to adapt to the new level of light. Explain how all nerves are like this. Our taste buds, too, take some time to adjust to new flavors. Our taste buds are accustomed to being bombarded with highly palatable foods, due to our “obesogenic” environment. Explain that the thing with taste buds is that they take several weeks to change. However, cravings are temporarily and will subside with time.
- If cravings are persistent, or are de-railing a participant’s efforts, discuss healthy ways to satisfy desires for sweets (e.g. banana ice-cream, frozen grapes) and savory foods (e.g. homemade chips or onion rings).

*Concerns about specific nutrients.*

Assure participants that reputable sources (e.g. the American Dietetic Association) have approved well-planned vegetarian diets as appropriate for individuals during all stages of life. Validate that this is a perfectly natural concern, as the whole foods plant-based diet is likely a foreign way of eating, and that humans have evolved to be attentive to issues of scarcity. Assure participants that most people in modern environments do not suffer from deficiencies/food scarcity, but rather excess. Forward participants to [nutritionfacts.org](file:///C:\Users\chris\Dropbox\Drafts%20-%20Christina%20Chwyl\thesis\nutritionfacts.org) for reliable, evidence-based nutrition information, as well as further reading on plant-based sources for various nutrients.

*Lack of knowledge on how to adapt preferred or traditional dishes to be whole foods plant-based*

Coaches will work with participants to find substitutions and alternate cooking methods so that they can adapt favorite, preferred and/or traditional dishes to be whole foods plant-based. Coaches will work with participants to ensure that the whole foods plant-based diet can fit with their unique lifestyle and needs.

*Miscellaneous.*

- If participants are struggling with a specific area that is covered in materials from a later week (e.g. social support, small weight gain), it might be appropriate to point participants towards those materials during or after the call. Be careful not to “information overload” participants; only send extra materials if participants indicate to you that this would be helpful, or if what you covered during the phone coaching call was not sufficient to address their area of concern.

Next, provide a brief summary of the week’s content:

- *Content Overview:* e.g*. “This week, the module reviewed calorie-density, and how to select foods that are high in water and fiber that will keep us full. Do you have any questions about the material?”*

Next, briefly review participants’ worksheet(s):

- *Worksheet Check-In: “I see from your worksheet that you were going to try a whole foods plant-based version of one of your favorite foods—lasagna—this past week. That sounds delicious! How did it go?”*

Answering Questions about Non-Study-Related Weight loss Components

In coaching calls, it is possible that participants will ask about weight loss behaviors that are not components of the intervention.

If participants ask about physical activity:

- Explain that we did not cover this in the materials because sometimes it is easiest for people to focus on changing one behavior at a time. Provide a neutral, non-leading response. Discuss how physical activity is beneficial for health, and that some participants may opt to start/change their physical activity routine while enrolled in the study, while others may opt to start/change their physical activity routine after they have finished the study and/or have a firm handle on whole foods plant-based eating. Either approach is okay and that it depends upon the specific person and their goals.

If participants ask whether it is okay to track what they eat (or if you realize that a participant has been tracking their calories):

- Explain that calorie-tracking is not the approach we adopt in this study, because many people do not find it sustainable long-term. If participants are insistent upon dietary tracking, provide them with a simple template for tracking dietary intake (but not calorie intake) as follows:

|  | Sun | Mon | Tues | Wed | Thurs | Fri | Sat |
| --- | --- | --- | --- | --- | --- | --- | --- |
| **Green Zone Foods** (starches, potatoes, whole grains, beans, lentils, vegetables, fruit, berries) |  |  |  |  |  |  |  |
| **Orange Zone Foods** (sugar, alcohol, tofu, tempeh) |  |  |  |  |  |  |  |
| **Red Zone Foods** (oil, meat, cheese, butter, fish, chicken) |  |  |  |  |  |  |  |

If Participants Continue to Struggle to Adhere to a Whole Foods Plant-Based Diet

- Most participants will do best with clear, unambiguous “black and white” categories of what they can and cannot eat (i.e., green, orange, and red-zone foods). Tell participants that for the duration of the study, we ask that participants do their best to closely stick to these categories—greater weight loss will follow greater adherence. Tell participants that the more closely they can stick with the diet, the easier it will be for them in the long-term (see “cravings” section above).
- [If unable to stick to Traffic Light Diet System one-week after the 2-week transition period]: say to give it their best go another week.

[If unable to stick to Traffic Light Diet System two-weeks after the 2-week transition period]: Start with one whole foods plant-based meal a day (breakfast is typically easiest). In each subsequent week, aim to increase the percentage of whole foods plant-based eating, one step at a time (e.g., reduce amount of oil while cooking, swap meat in a sandwich with hummus, swap snack of cheese and crackers with fruit and low-fat dip).

| *Supplementary Table S1.* Summary of Intervention Changes Following the Pretest | |
| --- | --- |
| **Modification Rationale** | **Modification Description** |
| Confusion about permissible foods in a WFPBD (oil, sugar, salt, pasta, bread) | - Shifted content on oil, sugar, salt, pasta, and bread to be presented in weeks 1 and 2 |
| Sub-optimal weight loss (treatment did not produce 5% weight loss for most participants) | - In initial phone check-ins, established program weight loss goal - Included goal weight on weight chart for visual reminder |
| Confusion on dietary choices underlying weight plateaus | - In phone check-ins, asked more specific dietary adherence questions (e.g., structured dietary recall questions) |
| Low module acceptability (mean acceptability < 4) on weeks 3 and 10 | - Streamlined content to reduce module length - Made non-essential materials optional - Added pandemic-friendly grocery shopping tips (e.g., online orders) |
| Improve content’s representativeness | - Included greater diversity of body sizes in program materials |
| Improve user experience | - Created mobile-friendly and printable pdfs of key materials - Provided links to transcripts of videos - Included guide on accessing subtitles on YouTube |
| Technical Confusions | - Added Google Classrooms tutorial to baseline assessment - Improved handout formatting |

*Supplementary Table 2.* Behavioral Change Clusters (BCT) and Techniques Used in the Intervention

| **BCT Cluster** | **BCT Technique** | **Where Technique is Used in Intervention** |
| --- | --- | --- |
| Goals and Planning | Goal setting (behavior) | - Agree on daily eating plan (foods to consume, limit and avoid) |
|  | Problem solving | - Relapse prevention module  - Problem-solving module  - Individualized problem-solving support in coaching calls |
|  | Goal setting (outcome) | - Individualized weight loss goal set at start of program (in Phase 2) |
|  | Action planning | - Participants prompted to create detailed grocery shopping and meal preparation plans |
|  | Review behavior goal(s) | - Eating behavior goals continually monitored with participants and adjusted as needed |
|  | Discrepancy between current behavior and goal | - Gap between eating behaviors and weight outcomes discussed in coaching calls |
|  | Review outcome goal(s) | - Weight goals modified as needed |
| Feedback and Monitoring | Feedback on behavior | - Eating behaviors discussed in each coaching call; with feedback provided on areas to change |
|  | Self-monitoring of outcome(s) of behavior | - Participants self-weighed weekly |
|  | Feedback on outcome(s) of behavior | - Feedback provided on weight trajectory in coaching calls |
| Social Support | Social support  (unspecified) | - Participants provided with resources on where to find support with a WFPBD in their community  - Participants encouraged to build a social support network to assist with adopting and sustaining a WFPBD |
| Shaping Knowledge | Instruction on how to perform a behavior | - Cooking videos included in web-modules |
|  | Information about antecedents | - Participants encouraged to identify antecedents of emotional eating and dietary lapses through “chain” analyses |
|  | Re-attribution | - Participants taught to attribute overweight/obesity to food environment, as opposed to internal factors (e.g., willpower, lack of self-control) |
| Natural Consequences | Information about health consequences | - Relationship between diet, overweight and health outcomes discussed |
| Comparison of Behavior | Demonstration of the behavior | - Examples of individuals who have successfully adopted a WFPBD provided throughout intervention (“Plant-powered Paragons”) |
| Associations | Demonstration of the behavior | - Participants encouraged to place healthy foods in prominent and visible locations (e.g., fruit on counter, healthy food at the front of the fridge) |
|  | Remove access to the reward | - Participants encouraged to remove accessibility of non-WFPBD foods |
| Repetition and Substitution | Behavioral practice/rehearsal | - Participants practiced ahead of time how to explain their diet and pressures to consume non-WFPBD foods in social situations |
|  | Habit formation | - Participants prompted to identify cues and rewards for dietary behaviors that they want to become habitual, and to repeat these behaviors |
|  | Graded tasks | - Participants make small changes to transition to a WFPBD prior to fully trialing a WFPBD |
| Comparison of outcomes | Credible source | - Videos from WFPBD experts presented in web-modules |
|  | Pros and Cons | - A motivational interviewing approach was used for phone coaching calls, including assisting individuals in identifying pros and cons of changing their diet |
| Reward and Threat | Social reward | - Positive reinforcement provided from phone coach for progress in dietary modification and weight loss |
| Regulation | Conserving mental resources | - Participants encouraged to make a priori decisions about eating to decrease decision-making fatigue |
| Antecedents | Restructuring the physical environment | - Stimulus control emphasized, including cleaning out pantry and making unhealthy foods less readily accessible |
|  | Avoidance/reducing exposure to cues for the behavior | - Advised on how to avoid exposure to social and environmental cues that trigger cravings or desires to eat non-WFPBD foods |
| Identity | Identification of self as role model | - Participants encouraged to consider how their healthy eating might set a good example to those in their family and community |
|  | Framing/reframing | - Participants taught to identify, and restructure distorted thinking (e.g., black and white thinking) that interfere with dietary adherence and staying on track |
|  | Identity associated with changed behavior | - Participants encouraged to identify as a healthy eater |
| Self-belief | Focus on past success | - Participants encouraged to reflect on past successes losing weight and eating healthfully |
|  | Self-talk | - Participants encouraged to adopt a self-compassionate stance towards setbacks |

*Note.* The primary investigator coded the intervention according to Michie’s Behavior Change Technique (BCT) v1 taxonomy (Michie et al., 2013).

| Supplementary Table 3. Intervention Outline | | |
| --- | --- | --- |
| **Week** | **Topic** | **Content Outline** |
| **Part 1: Transition** | | |
| 1 | Introduction to the Program and a WFPBD | - Program Principles and Overview - Introduction to the Traffic Light Diet System - Energy Density |
| 2 | Plant-Based Health and Eating 101 | - Health Benefits of a WFPBD - Typical WFPBD meals - Kitchen and Pantry Essentials |
| **Part 2: Change** | | |
| 3 | Grocery Shopping | - Optimizing your Home Food Environment - Grocery Shopping |
| 4 | Changing Tastes and Substitutions | - Psychoeducation on Cravings - Urge Surfing - Substituting non-WFPBD ingredients in cooking and baking |
| 5 | Holidays, Travel, and Eating Out | - Identifying motivations underlying health behavior change - Tips and tricks for eating out, travelling, and holidays and special occasions |
| 6 | Keys to Success | - Simple meal guidelines - Maintaining motivation - The importance of regular self-weighing - *Optional:* The metabolism of carbohydrates and fats |
| **Part 3: Sustain** | | |
| 7 | Why Whole Food Plant-Based and Advanced Cooking Tips | - Research on low-carbohydrate diets - Blue zones - Salt and sugar - Advanced cooking techniques & fruit and vegetable storage |
| 9 | Navigating Social Situations | - Decreasing unhelpful and increasing helpful social cues - Navigating social situations when plant-based - *Optional:* Plant-Based Kids |
| 9 | Emotional Eating | - Strategies for handling emotional eating - Healthy substitutions for common cravings |
| 10 | Simplifying Decision-Making | - Decreasing decision-making fatigue and habit formation - Choosing your approach to a WFPBD - *Optional:* Problem-solving |
| 11 | Handling Setbacks | - Creating a ‘Get Back on Track’ plan - Challenging cognitive distortions - Practicing self-compassion - The Pleasure Trap |
| 12 | Celebrating Accomplishments & Thinking Long-term | - Reflecting on Accomplishments - Challenging black and white & permission-giving thoughts - Content review |
